# Sandal reactive dyes decolorization and cytotoxicity reduction using manganese peroxidase immobilized onto polyvinyl alcohol-alginate beads

**DOI:** 10.1186/s13065-015-0125-0

**Published:** 2015-09-15

**Authors:** Muhammad Bilal, Muhammad Asgher

**Affiliations:** Industrial Biotechnology Laboratory, Department of Biochemistry, University of Agriculture, Faisalabad, Pakistan

**Keywords:** Manganese peroxidase, *Ganoderma lucidum*, Immobilization, Sandal reactive dyes, Textile wastewater, Decolorization, Cytotoxicity

## Abstract

**Background:**

Fungal manganese peroxidases (MnPs) have great potential as bio-remediating agents and can be used continuously in the immobilized form like many other enzymes.

**Results:**

In the present study, purified manganese peroxidase (MnP) enzyme isolated from *Ganoderma lucidum* IBL-05 was immobilized onto polyvinyl alcohol-alginate beads and investigated its potential for the decolorization and detoxification of new class of reactive dyes and textile wastewater. The optimal conditions for MnP immobilization were 10 % (w/v) PVA, 1.5 % sodium alginate, 3 % boric acid and 2 % CaCl_2_ solution. The optimum pH, temperature and kinetic parameters (*K*_*m*_ and *V*_*max*_) for free and immobilized MnP were found to be significantly altered after immobilization. The immobilized MnP showed high decolorization efficiency for Sandal reactive dyes (78.14–92.29 %) and textile wastewater (61–80 %). Reusability studies showed that after six consecutive dye decolorization cycles, the PVA coupled MnP retained more than 60 % of its initial activity (64.9 % after 6th cycle form 92.29 % in 1st cycle) for Sandal-fix Foron Blue E_2_BLN dye. The water quality assurance parameters (BOD, COD and TOC) and cytotoxicity (haemolytic and brine shrimp lethality tests) studies before and after treatment were employed and results revealed that both the dyes aqueous solution and textile wastewater were cytotoxic that reduced significantly after treatment.

**Conclusions:**

The decolorization and cytotoxicity outcomes indicated that immobilized MnP in PVA–alginate beads can be efficiently exploited for industrial and environmental applications, especially for remediation of textile dyes containing wastewater effluents.

**Graphical abstract:**
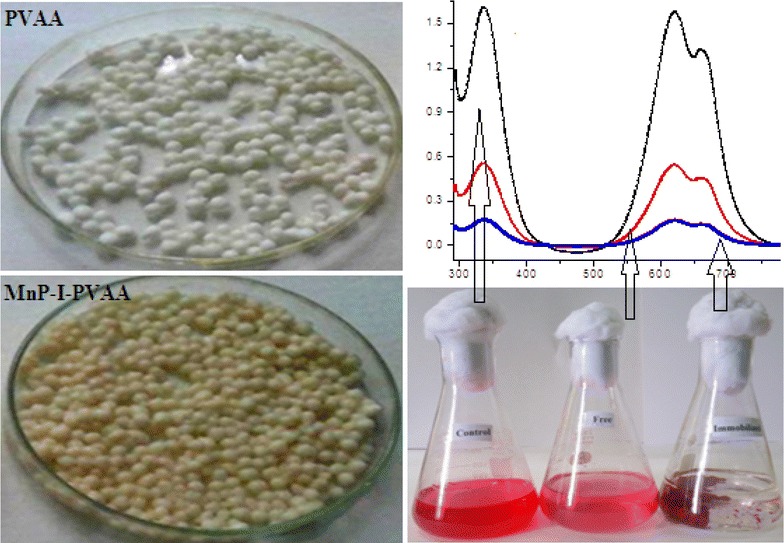
Dye decolorizing potential of immobilized MnP

## Background

No doubt, industrial establishment is necessary for growth, but it always associated with a cost paid in terms of pollution (air, water, soil). The rapid stride in textile industry is one of the major concerns to release toxic chemicals into the environment, especially toxic dyes [1–3]. Reports showed that more than 10,000 different types of dyes with an estimated annual production of 7.105 metric tons are commercially available worldwide [4]. Other than textile industries, dyes are also used in paper and pulp, dye intermediates, pharmaceutical and tannery etc. [3]. Among dyes, reactive dyes are extensively used, fundamentally due to the capacity of their reactive groups to bind on fibers. The discharge of dye-containing effluents into the water bodies is undesirable, because many of dyes are toxic, carcinogenic or mutagenic in nature. Among the damages caused, genotoxic and mutagenic effects have shown to be worrying, due to their capacity to induce genetic damage, which can lead to several health problems and also affect future generations [2].

Due to very stable and xenobiotic nature, reactive dyes are difficult to degrade or exhibit slow degradation [5]. In literature, various physical/chemical methods have been reported by several authors to effectively process the textile effluents such as adsorption, precipitation, chemical reduction, ionizing radiations and ultrafiltration. However, these tested methods are not economically acceptable for application to large-scale effluents due to many drawbacks, such as high operating and equipment costs, low efficiency, secondary pollution, residues waste problems and the inability to treat a wide array of dyes having structural diversity [1, 4]. Although these methods are employed to decolorize the dyes, their capacity to reduce the toxicity is still a matter of major concern. These facts certainly demand the development of an efficient, cost effective and green technique for the detoxification and decolorization of dyes [1]. Biodegradation of toxic dyes is considered best option in this regard and is a key research area in the environmental sciences [6–11].

An advantage of enzymatic approach to treat dye is that enzymes can react with variety of compounds, fast decolorization rate and removal is quite fast at fixed conditions [5]. The major reason that enzymatic treatments have not yet been applied on an industrial scale is due to the huge volume of polluted wastewater demanding remediation. Free enzymes suffer certain drawbacks such as thermal instability, reusability, susceptibility to attack from proteases, activity inhibition and are also sensitive to temperature and pH [6–9, 12–19]. To overcome limitations of free enzymes, the use of immobilized enzyme on various supports has been suggested due to its stability and reusability and previously reported studies regarding dye decolorization highlighted the importance of immobilization since encouraging results were obtained [5, 7, 8, 10, 11, 20–25].

Manganese peroxidase (MnP) is a versatile biocatalyst that gained significant importance in bioremediation, biomass delignification, chemical synthesis, bio-pulping and biosensor development, textile finishing and wine stabilization [5]. *Ganoderma lucidum* is potent lignin degrading white rot fungus (WRF), able to grow on variety of wastes and have been intensively studied so far to degrade a wide range of pollutants [5]. However, to best our knowledge, the direct application of ligninolytic enzymes for decolorization of textile dyes, especially Sandal reactive dyes has not yet been tried. Sandal dyestuff industries Ltd (Sandal Group) have main offices in Bangladesh and Faisalabad (Pakistan) and has been the agent of BASF AG, Germany, Bayer AG, Germany and Sandoz, Switzerland for their disperse and reactive dyes, but now Sandal, started reactive dyes production locally, and these reactive dyes are exporting to Turkey, Italy, Egypt, Spain, Bangladesh, Morocco, South Africa, Malaysia and Australia.

Therefore, the purpose of the present study was to develop an effective method for immobilization of MnP onto PVA and to decolorize an array of synthetic reactive dyes and real textile wastewater. The treatment efficiency was evaluated on the basis of decolorization, water quality assurance parameters (BOD, COD, TOC, pH) and cytotoxicity (erythrocytes lysis and brine shrimp lethality) reduction. Upon completion of synthetic dye decolorization investigation, wastewater was collected from ten local textile units and tested for decolorization and cytotoxicity reduction by MnP-I-PVAA and F-MnP for the generalization of results.

## Experimental

### Chemicals and reagents

Coomassie Brilliant Blue G-250, sodium dodecyl sulphate, Sephadex G100, *N*, *N*, *N*′, *N*′-tetra-methyl ethylene diamine, *ß*-mercaptoethanol, trizma base, polyvinyl alcohol (*M*w = 124–186 kDa), sodium alginate, calcium chloride and boric acid were supplied by Fluka-Sigma-Aldrich (USA) and standard protein markers were purchased from Fermentas (U.K). Triton X-100 and cyclophosphamide were provided by Merck (Germany) and Scharlau (Spain). All other chemicals used in this work were of analytical grade and used without further purification.

### Collection and preparation of substrate

Agricultural waste, wheat bran used as growth substrate was collected from Students Farm, University of Agriculture, Faisalabad, Pakistan, washed, sliced, sun dried following oven drying at 60 °C to constant weight. The dried substrate was pulverized to 0.45–0.90 mm (20–40 meshes) by grinder (Ashraf Herbal Laboratories, Faisalabad) and stored in airtight plastic jars.

### Fermentative organism and inoculum development

A pure culture of an indigenous fungal strain *G. lucidum* IBL-05 available in Industrial Biotechnology Laboratory, Department of Biochemistry, University of Agriculture; Faisalabad was raised on potato dextrose agar (PDA) slants at pH 4.5 and 30 °C. Kirk’s basal medium along with 1 % (w/v) Millipore filtered sterile glucose solution was used as inoculum medium. The medium pH was adjusted to 4.5 (HCl/NaOH), sterilized (Sanyo, Japan) at 121 °C and 15 psi for 15 min. *G. lucidum* IBL-05 spores were transferred to the sterilized inoculum medium under sterile conditions in laminar flow (Dalton, Japan). The inoculated flask was incubated at 30 °C for 5 days in an orbital shaker (Sanyo-Gallenkamp, UK) with continuous shaking (120 rpm) to obtain homogenous spore suspension (~1×10^7^ spore/mL), counted by hemocytometer (Neubauer counting chamber) (Sigma-aldrich, USA) [5].

### Solid state fermentation for enzyme production

Cotton plugged Erlenmeyer flasks (triplicate), containing 5 g wheat bran were moistened with Kirk’s basal nutrient medium (66 % w/w) at pH 4.5. The flasks were sterilized, inoculated with 5 mL (1 × 10^7^ spores/mL) freshly prepared *G. lucidum* IBL-05 spore suspension and placed at 30 °C for fermentation in still culture incubator (Sanyo, Japan) for 5 days. After stipulated time period, 100 mL of distilled water was added into the fermented flasks, shaken for 30 min at 120 rpm (Sanyo-Gallenkamp, UK), filtered, centrifuged (Eppendorf 5415C, Germany) and clear supernatant thus obtained was subjected to ligninolytic enzymes estimation [23] and purified further.

### Purification of MnP

Four step purification procedure involving ammonium sulphate fractionation, dialysis, DEAE-cellulose ion exchange and G-100 Sephadex gel permeation chromatography was gradually employed for the purification of MnP. Briefly, crude MnP extract obtained from 5 days old culture of *G. lucidum* IBL-05 was centrifuged at 3000×*g* for 15 min to increase clarity using Eppendorfs centrifuge machine (Centrifuge 5415 C, Germany). The supernatant/filtrate was saturated (up to 35 %) by gradual addition of ammonium sulphate, kept overnight at 4 °C and precipitates, thus obtained were recovered (10,000 rpm for 20 min at 4 °C) and supernatant was again saturated by adding ammonium sulfate (up to 65 %), allowed to stand overnight at 4 °C, centrifuged and pellets were dissolved in 50 mM Sodium malonate buffer (pH 4.5), dialyzed and finally, dialyzate was freeze dried. The dialysate obtained was subjected to ion exchange chromatography using diethyl amino ethyl (DEAE) cellulose column. The column was equilibrated with phosphate buffer (pH 6.5) for 24 h and eluted with 0–1.0 M linear gradient of NaCl in 50 mM Na-malonate buffer at a flow rate of 0.5 mL/min. A total 60 fractions, each 1.5 mL were collected and analyzed for enzyme activity and protein contents. The MnP active fractions were concentrated and loaded onto Sephadex-G-100 column (10 × 300 mm). A 50 mM malonate buffer was used for elution at a flow rate of 0.3 mL/min and active fractions (up to 30, 1 mL) were collected and absorbance was measured at 280 nm (CE Cecil 7200, Germany). The purified and concentrated enzyme was stored at −20 °C.

### Immobilization of MnP

The PVA solution (8–12 %) and Na-alginate (0.5–4.0 %) were heated at 60 °C to dissolve properly, cooled and mixed with 10 mL of MnP solution. The resultant mixture was extruded drop wise into boric acid (3 % v/v) and CaCl_2_ (2 % w/v) solution using syringe (nozzle diameter 5 mm) needle to form uniform size beads and stirred for 30–50 min to solidify, thoroughly washed with distilled water and store at 4 °C for 24 h [26]. The immobilization percentage was calculated by relation Eq. (1).

1 $$ {\text{Immobilization }}({\text{\% }}) = \frac{\text{Total activity of immobilized enzyme}}{\text{Total activity of free enyme}} \times 100. $$

### Effect of pH, temperature and substrate concentration on immobilization

In this set of experiment, the MnP-I-PVAA activities were tested at different pH values (3–10). The buffers used were (0.2 M): tartrate buffer, pH 3.0; sodium malonate buffer, pH 4.0; citrate phosphate, pH 5.0 and pH 6.0; sodium phosphate, pH 7.0 and 8.0; carbonate-bicarbonate buffer, pH 9.0 and pH 10.0, whereas temperature effect was checked by incubating the enzyme at varying temperature (30–70 °C) for 1 h at optimum pH before running the routine MnP assay. The effect of substrate concentration was studied by varying the substrate MnSO_4_ (0.1–1 mM) at optimum pH and temperature. Lineweaver–Burk’s reciprocal plots were constructed between 1/S and 1/V_0_ and kinetic parameters of Michaelis–Menten (*K*_m_ and *V*_max_) were calculated [23].

### MnP assay and protein estimation

For MnP activity determination, 1 mM MnSO_4_ (1 mL), 50 mM sodium malonate buffer (1 mL, pH 4.5) and enzyme solution (0.1 mL) were thoroughly mixed, followed by the addition of 0.1 mM H_2_O_2_ (0.5 mL) as reaction initiator and absorbance was checked at 270 nm (ε_270_ = 11570 M cm^−1^) with 10 min interval. For protein estimation, Bradford reagent (1 mL) was added into 100 μL of each solution, mixed well (vortex mixer) and ΔA was monitored at 595 nm (UV double beam spectrophotometer (HALO DB-20) [27].

### Dyes and wastewater collection

Five Sandal Reactive dyes namely Sandal-fix Red C_4_BLN, Sandal-fix Turq Blue GWF, Sandal-fix Golden Yellow CRL, Sandal-fix Black CKF and Sandal-fix Foron Blue E_2_BLN were gifted by Dr. Shaukat Ali (consultant-Sandal dye stuffs), Color and Dyes Laboratory, University of Agriculture, Faisalabad, Pakistan. Selected dyes characteristics are shown in Table 1. The textile wastewater sample were collected from Rashid Textile, Arif Textile, Yasser Textile, Mian Chemicals, Taj Mahal Printing, Bashir Printing, M. Shafique Textile Mill, Lili Printing, Amar Bilal Textile Mill, Ali Hajveri Printing industries (numbered 1-10, respectively), located in different areas of Faisalabad, Pakistan.

**Table 1:** Characteristics of Sandal reactive dyes used in present investigation

Dyes	Color	$\lambda_{ \hbox{max} }$	Class	CI number
Sandal-fix Red C_4_BL	Red	540	Reactive	Reactive Red 195A
Sandal-fix Turq Blue GWF	Blue	664	Reactive	Reactive Blue 21
Sandal-fix Golden Yellow CRL	Yellow	414	Reactive	Reactive Yellow 145A
Sandal-fix Black CKF	Black	598	Reactive	Mixture
Sandal-fix Foron Blue E_2_BLN	Foron Blue	560	Reactive	Not known

*CI* color index

### Decolorization of Sandal reactive dyes and textile wastewater

A set of Sandal reactive dyes was selected to investigate the decolorization potential of MnP-I-PVAA versus F-MnP. For this, the MnP-I-PVAA and F-MnP were transferred to 250 mL cotton plugged Erlenmeyer flasks (triplicate) containing 100 mL of individual dye solution (0.1 mg/mL), prepared in 50 mM sodium malonate buffer (pH 4.5). Flasks were incubated at 30 °C on rotary shaker (Sanyo-Gallenkamp, UK) at 150 rpm for 12 h. After designated time, the flasks contents were filtered, centrifuged (8000×*g*, 10 min) and residual dye concentration was monitored at respective wavelengths (HALO DB-20).The decolorization efficiency of MnP-I-PVAA and F-MnP for each dye was calculated using relation in Eq. (2). Where A*i* and A*t* are representing absorbance at zero and time *t*. For the decolorization of real textile wastewater samples, same procedure was adopted and before treatment, the samples were filtered to remove debris and suspended particles.

2 $$ {\text{Decolorization }}({\text{\% }}) = \frac{{{\text{A}}i - {\text{A}}t}}{{{\text{A}}i}} \times 100 $$

### Reusability studies

The MnP-I-PVAA reusability efficiency for the decolorization was monitored up to six cycles. For this, the beads were incubated (Sanyo, Japan) with 1 mM MnSO4 in respective dye solution (0.1 mg/mL) prepared in 50 mM sodium malonate buffer of pH 4.5 at 30 °C for 12 h. At the end of each cycle, the beads were filtered and washed three times with the sodium malonate buffer.

### Water quality assurance parameters analysis

The water quality assurance parameters such as BOD, COD, TOC and pH were measured where maximum decolorization was achieved both for synthetic dye solution and textile wastewater. BOD, COD and pH values were measured using BOD, COD and pH meters (Lovibond, water testing systems). For TOC measurement, 2 N K_2_Cr_2_O_7_ (1 mL) and H_2_SO_4_ (1.6 mL) were taken in digestion flask containing dyes samples (4 mL) and the contents were digested for 1.5 h at 110 °C, cooled and absorbance was monitored at 590 nm (HALO DB-20).

### Cytotoxicity evaluation

To evaluate the MnP-I-PVAA and F-MnP effect on cytotoxicity reduction, erythrocytes lysis and brine shrimp lethality tests were used [2]. The cytotoxicity of samples was measured where maximum decolorization was achieved both for synthetic dye solution and textile wastewater samples.

## Results and discussion

### MnP production and purification

An indigenous WRF strain, *G. lucidum* IBL-05 was exploited to produce MnP in solid substrate fermentation (SSF) medium of wheat bran at pre-optimized conditions (moisture, 50 %; substrate, 5 g; pH, 5.5; temperature, 30 °C; carbon source, 2 % glucose; nitrogen source, 0.02 % yeast extract; C: N ratio, 25:1; fungal spore suspension, 5 mL, 5 days fermentation time). A quite significant MnP activity of 717.7 ± 2.3 U/mL was recovered in the culture filtrate after 5 days incubation. The cell-free crude MnP extract thus produced was completely salted out at 65 % saturation with (NH_4_)_2_SO_4_ to 1.73 fold purification with specific activity of 273.01 U/mg. After ion exchange and sephadexG-100 gel filtration chromatography, the specific activity of MnP increased to 539.59 U/mg with 3.43 folds purification. The summary of MnP purification results are presented in Table 2. The purification trend was found comparable with earlier studies e.g. MnP from *S. commune* IBL-06 was salted out at 55 % (NH_4_)_2_SO_4_ saturation with 1.8 fold purification and specific activity of 394 U/mg [23]. Fractionation of MnP from *Phanerochaete chrysosporium* by DEAE Sepharose, followed by ion-exchange and UltragelAcA54 gel filtration chromatography resulted in 23.08 % activity yield with 5.8 fold purification [28].

**Table 2:** Purification summary of MnP produced by *G. lucidum* IBL-05 in solid state fermentation

Purification Steps	Total volume (mL)	Enzyme activity (U/mL)	Protein contents (mg/mL)	Specific activity (U/mg)	Purification fold
Crude extract	300	717.4	4.34	132.32	1
Ammonium Sulphate ppt.	22	613.7	2.45	273.01	1.73
Dialysis	21	598.09	2.13	390.91	1.95
DEAE-cellulose	12	584.3	1.68	420.34	2.78
Sephadex G-100	9	569.6	0.9	539.59	3.43

### Optimal immobilization of MnP

**Fig. 1:**
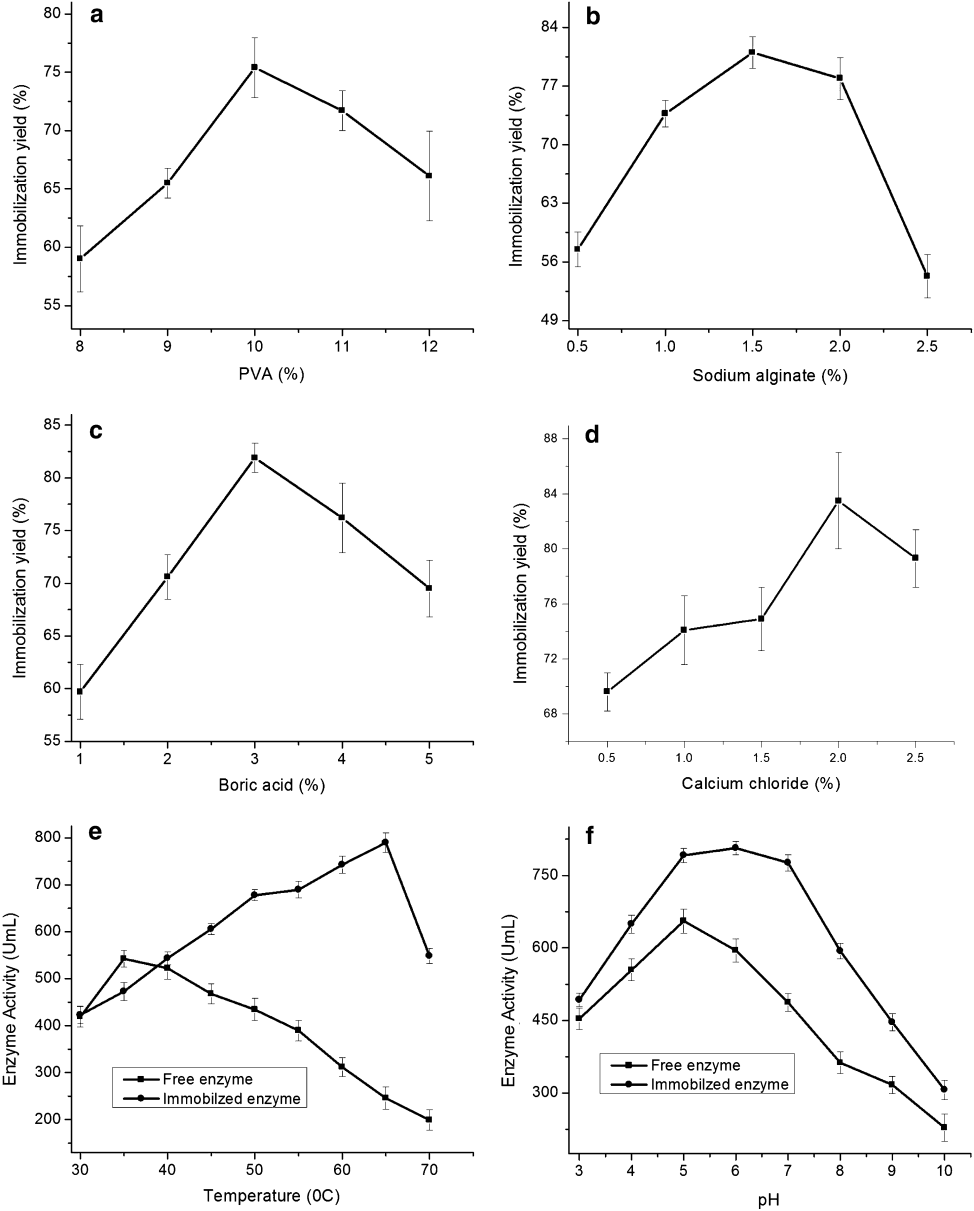
MnP immobilization yield: **a** PVA concentration effect, **b** sodium alginate, **c** boric acid **d** calcium chloride and **e** temperature effect on F-MnP and MnP-I-PVAA activities, **f** pH on F-MnP and nP-I-PVAA activities

The purified MnP from *G. lucidum* IBL-05 was immobilized onto PVA–alginate beads and range of affecting variables such as concentration of immobilization matrix (PVA and Na-alginate) and cross-linkers (boric acid and calcium chloride) were studied (Fig. 1). During preparation of beads, different concentrations of PVA (8–12 %) were tried with the aim to acquire beads with desired mechanical strength. Beads developed using 10 % PVA were found to be most suitable for immobilization exhibiting highest immobilization yield (IY) (Fig. 1a). Below or above this optimal concentration, the immobilization efficiency declined that might be attributed to small pore size of the beads, restricting the diffusibility of the substrate. From the Fig. 1b, it can be observed that sodium alginate at a concentration of 1.5 % (w/v) registered the highest entrapped MnP activity. Low incorporation of Na-alginate at lower and higher concentrations leads to soft, unstable and flaccid beads formation and diffusional limitation of substrate into entrapped enzyme and thus, IY may reduce. Likewise, 3 % boric acid (Fig. 1c) showed better performance but higher concentration beyond this, diminished the IY, which could be due to denser beads formation and restricted substrate diffusion [26]. Furthermore, calcium chloride is used as a cross linking agent, and its concentration affects the activity and the stability of immobilized MnP. Figure 1d shows that calcium chloride at concentration of 2 % furnished better yield and low IY at lower concentration is due to unstable cross-linked gel formation and beads dispersion.

### Steady state kinetics

#### Optimum pH

The pH profile for soluble and immobilized MnP has been presented in Fig. 1f. It can be clearly observed that pH significantly affects the enzyme activity and pH values of 5.0 and 6.0 furnished highest activities for free and immobilized MnP, respectively. Unlike soluble MnP, the immobilized enzyme displayed good activity in broader pH range. It was reported that optimal pH may be altered by as much as 2 pH units. The shift in pH mostly depends on the pH of micro-environment around the immobilized enzyme and also the physico-chemical nature of support material contributes up to some extent. Similar findings were observed for immobilized phospholipase A1 in gelatin hydrogel [29], naringinase in PVA matrices [30], laccase in PVA cryo-gel carrier [31] and dextranase in bovine serum albumin [32].

#### Optimum temperature

The activity of free and immobilized MnP was checked as the function of varying temperature.

As depicted in Fig. 1e, the free and immobilized MnP activities were found higher at 35 and 65 °C, respectively, which indicates that immobilization, enables the MnP to be active and stable even at higher temperature and is credited to the PVA protective efficiency. The possible reason could be that some covalent linkages (hydrophobic and other secondary interactions of entrapped enzyme) developed, that might impair conformational flexibility requiring higher temperatures for the enzyme to reorganize and attain a proper conformation in order to keep its reactivity. Therefore, immobilized MnP opens the possibility to use it under environmental conditions at higher temperature. Similar observations were described previously [29, 33, 34].

#### *K*_m_ and *V*_max_

**Fig. 2:**
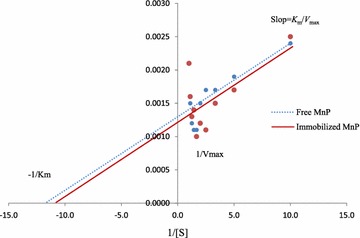
Lineweaver–Burk reciprocal plot: *K*_M_ and *V*_max_ for F-MnP and MnP-I-PVAA

The kinetic constants (*K*_m_ and *V*_mx_) of free and immobilized MnP were computed by intercepting the line on the X-axis and Y-axis of the Lineweaver–Burk plot, using MnSO_4_ as substrate. The highest activities of free and carrier bound MnP were found to be 859 IU/mL (*K*_m_ 65.5 mM and *V*_max_ 640 U/mL) and 896 IU/mL (*K*_m_ 70 mM and *V*_max_ 700 U/mL), respectively using non-linear regression analysis (Fig. 2). The change in kinetic parameters of the enzyme is a common manifestation during the process of immobilization. Attachment of enzyme molecules to the supporting matrix generates some hindrance which limits the accessibility of substrate to the enzyme and also the conformational changes in enzyme occurred after immobilization contribute to decreased affinity of enzyme for substrate [5]. Previously, Nunes and coworkers, [30] reported a higher Michaelis–Menten constant (K_M_) and lower maximum reaction velocity (*V*_max_) for immobilized naringinase than that of free enzyme. While the K_m_ values for immobilized lipase was found to be lower as compared to its free counterpart [35].

### Decolorization of Sandal reactive dyes and textile wastewater

**Fig. 3:**
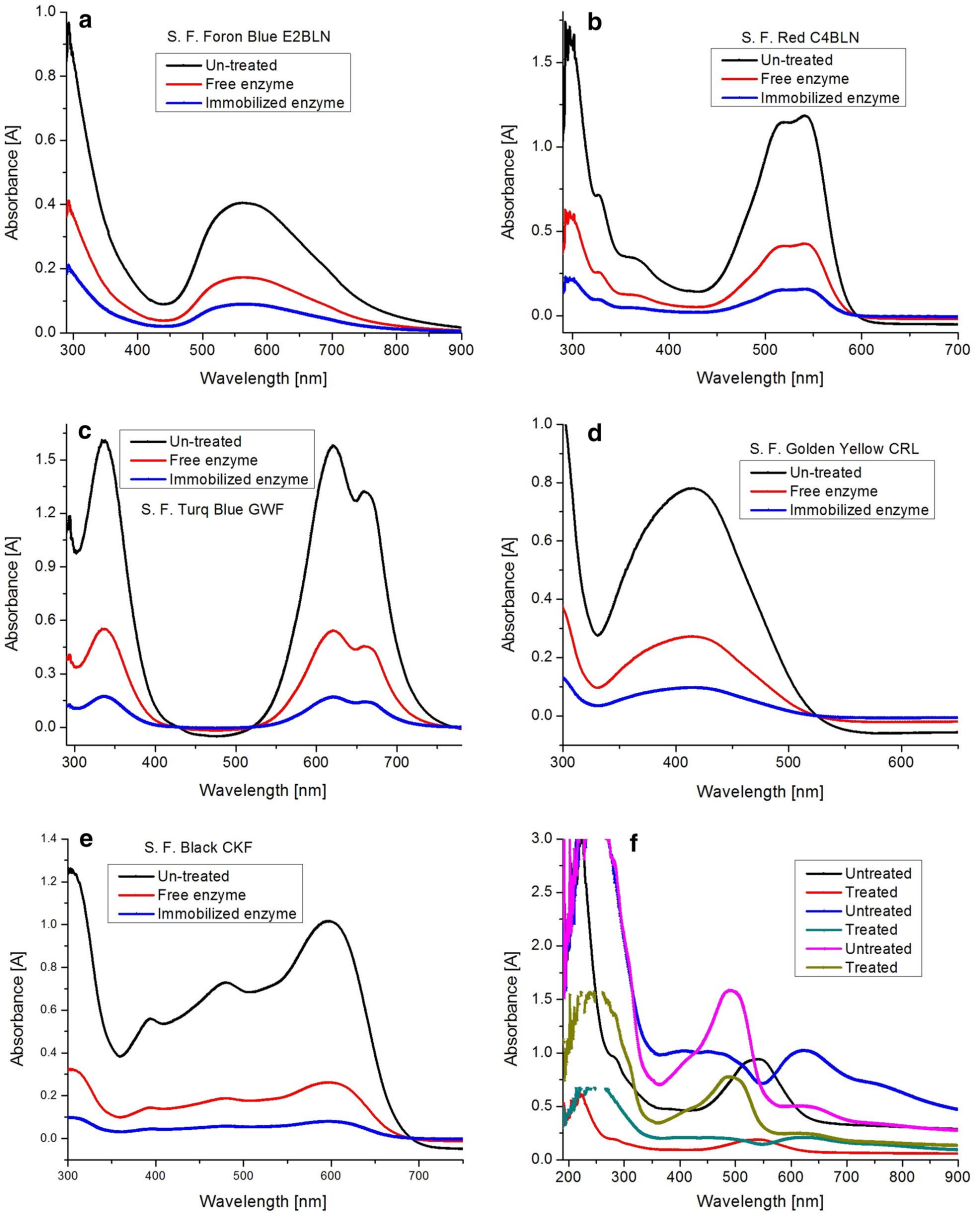
**a**–**e** UV–Vis spectra of sandal reactive dyes and **f** textile effluents (un-treated, F-MnP and MnP-I-PVAA treated)

**Fig. 4:**
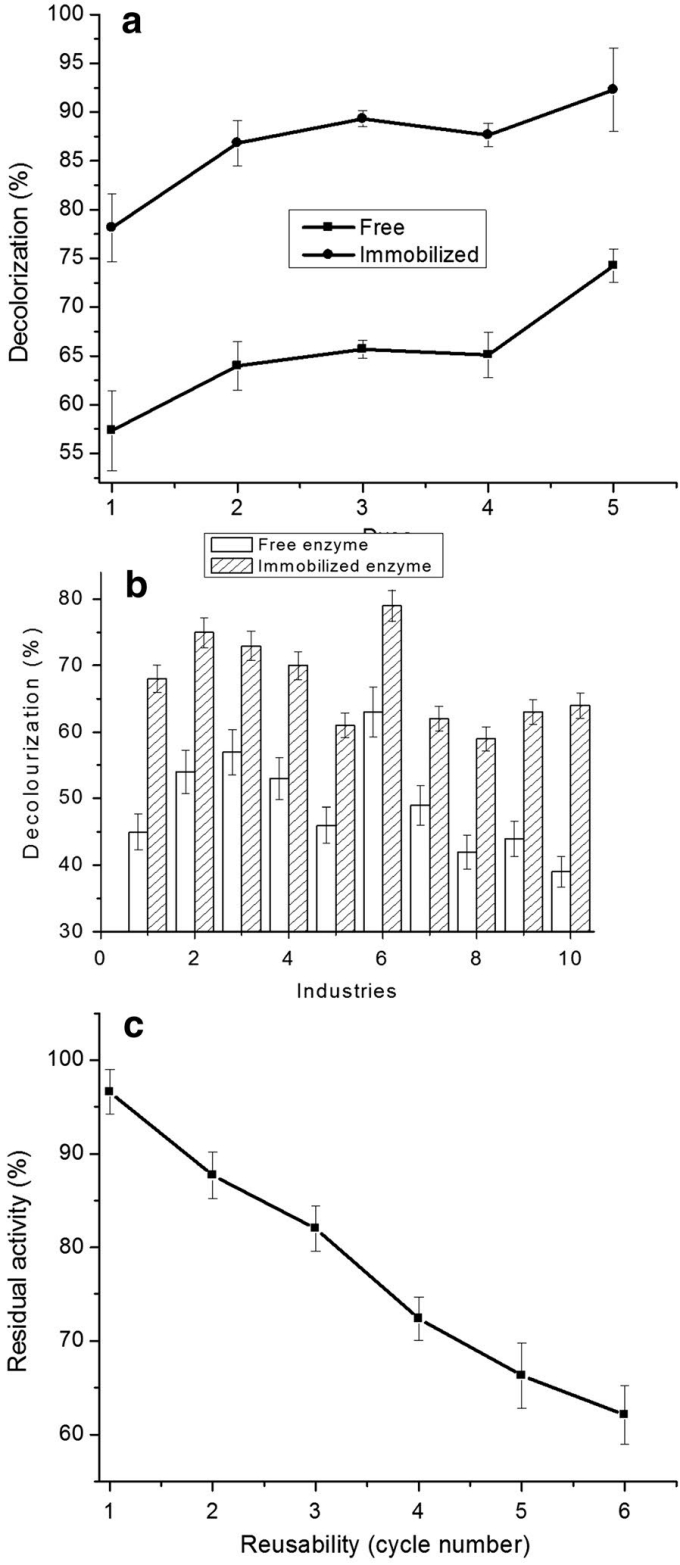
**a** Decolorization (%) Sandal reactive dyes (1–5) and **b** textile wastewater decolorization (%) treated by F-MnP and MnP-I-PVAA, **c** reusability of MnP-I-PVAA

The decolorization efficiencies (%) of F-MnP and MnP-I-PVAA were tested against set of Sandal reactive dyes and results, thus obtained are presented in Fig. 3a–e (UV–Vis spectra) and Fig. 4a (decolorization, %). From results, it was observed that immobilized MnP was more efficient for the decolorization of all investigated reactive dyes as compared to its free counterpart. In case of F-MnP, a decolorization of 57.34, 63.98, 65.66, 65.09 and 74.24 % of Sandal-fix Red C_4_BLN, Sandal-fix Turq Blue GWF, Sandal-fix Golden Yellow CRL, Sandal-fix Black CKF, Sandal-fix Foron Blue E_2_BLN, respectively were achieved, whereas it increased to 78.14, 86.81, 89.31, 87.63 and 92.29 %, respectively for PVA-I-MnP, which was 26.62, 26.30, 26.48, 25.72 and 19.56 % higher than F-MnP fraction for decolorization of Sandal-fix Red C_4_BLN, Sandal-fix Turq Blue GWF, Sandal-fix Golden Yellow CRL, Sandal-fix Black CKF, Sandal-fix Foron Blue E_2_BLN, respectively. Similarly, previous data reports regarding dye decolorization [6–8, 12, 15–19] revealed higher decolorization potential of immobilized MnP as compared to soluble MnP treatment. In case of MnP-I-PVAA, the decolorization efficiency was comparable with *P. chrysosporium* Ca-alginate beads [24], *Rhus vernificera*-poly (GMA/EGDMA) beads [25], *Pleurotus ostreatus*-sol‐gel, *G. lucidum*-Xero-gel [22] and Ca-alginate gel [8], which verified the dye remediation potential of PVA immobilized MnP. Upon completion of decolorization of synthetic dyes, same treatments were applied to investigate the decolorization efficiency for real textile wastewater, containing dyes mixture. Wastewater was collected from ten different textile units, treated by both F-MnP and MnP-I-PVAA systems and results are shown in Fig. 3f (UV–Vis representative spectra) and Fig. 4b (decolorization,  %). In case of F-MnP treatment, the decolorization was recorded in the range of 37–63 %, whereas it was 61–80 % for MnP-I-PVAA treatment. The decolorization efficiency for textile wastewater was slightly lower as compared to synthetic dyes aqueous solution and is attributed to the diverse nature of textile wastewater.

### MnP-I-PVAA reusability

To demonstrate the reusability properties of PVA entrapped MnP, beads were removed after stipulated reaction time (12 h) and washed thoroughly with distilled water. It was found that even after 6 consecutive dye decolorization cycles, the PVA coupled MnP retained more than 60 % of its initial activity (64.9 % after 6th cycle form 92.29 % in 1st cycle)for Sandal-fix Foron Blue E_2_BLNdye (Fig. 4c), however, for three cycles, the activity loss was insignificant and then gradually declined. This gradual decline in activity might be the result of plugging of the membrane pore and accumulation of high active free radicals e.g., Mn^3+^, lipid, hydroxyl, and peroxy-radicals and dimmer in the interior environment of each microsphere which entangled the active site of enzyme led to enzyme inactivation. Similar observations have been documented previously regarding reusability e.g. after 5 cycle, the dye removal efficiency of entrapped CBP-Con A complex was reduced to 67.9 % for DR19 and 63.5 % for dye mixture (DR19 + DB 9) [8] and 50 % loss of initial activity after five cycles for immobilized Horseradish peroxidase [8]. Results indicated that MnP immobilized onto PVA–alginate beads is a better choice with greater reusability efficiency for the removal of toxic pollutants/dyestuffs.

### Water quality assurance parameters

**Fig. 5:**
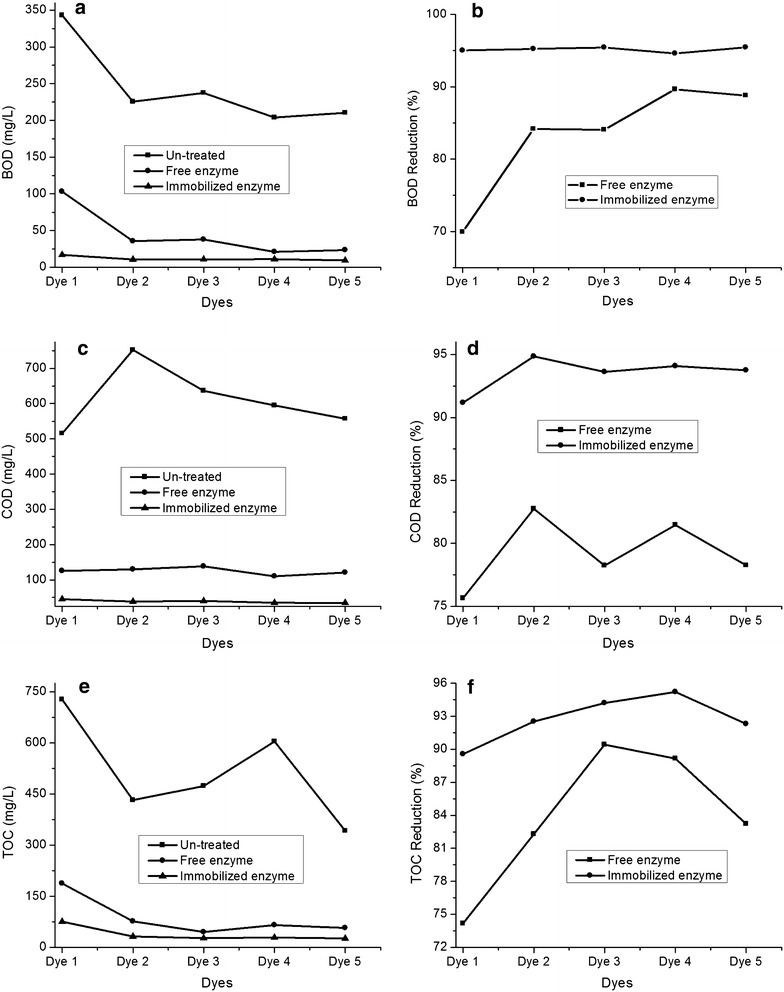
BOD, COD, TOC values of dyes [un-treated, F-MnP and MnP-I-PVAA treated (mg/L)] (**a**, **c**, **e**) and percentage reductions (**b**, **d**, **f**)

**Fig. 6:**
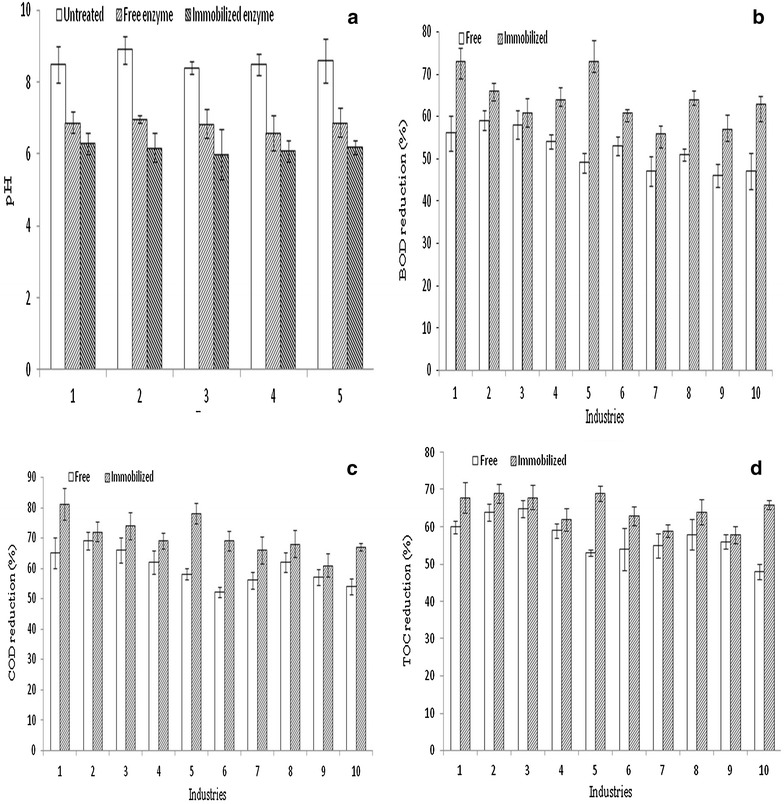
**a** Dyes solution pH (un-treated, F-MnP and MnP-I-PVAA treated), **b**–**d** percentage COD, BOD and TOC reductions of textile wastewater (F-MnP and MnP-I-PVAA treated)

The effect of treatment (F-MnP and MnP-I-PVA) for dye degradation was evaluated on the basis of BOD, COD, TOC, and pH, as shown in Fig. 5a–f. Before treatment, the BOD values were in the range of 204–343 mg/L of Sandal reactive dyes and reduced significantly after treatment. In case of F-MnP and MnP-I-PVAA treatments, the BOD values reduced to 21–103 mg/L and 10–17 mg/L, respectively. The percentage reductions in BOD values were 69.95, 84.16, 84.07, 89.66 and 88.77 % (F-MnP) and 95.02, 95.24, 95.44, 94.61 and 95.47 % (MnP-I-PVAA) for Sandal-fix Red C_4_BLN, Sandal-fix Turq Blue GWF, Sandal-fix Golden Yellow CRL, Sandal-fix Black CKF, Sandal-fix Foron Blue E_2_BLN, respectively. Before treatment, the COD was in the range of 515–752 mg/L and reduced to 110–138 mg/L and 34–45 mg/L for F-MnP and MnP-I-PVAA treated dyes. The percentage reductions were 75.65, 82.75, 78.23, 81.46 and 78.26 % (F-MnP) and 91.18, 94.85, 93.63, 94.09 and 93.75 % (MnP-I-PVAA) for Sandal-fix Red C_4_BLN, Sandal-fix Turq Blue GWF, Sandal-fix Golden Yellow CRL, Sandal-fix Black CKF, Sandal-fix Foron Blue E_2_BLN, respectively. The TOC values before treatment were in the range of 342–728 mg/L and reduced to 45–187 mg/L (F-MnP) and 32–75 mg/L (MnP-I-PVAA) after treatment. The percentage reductions in TOC were 74.18, 82.3, 90.43, 89.16, 83.23 % (F-MnP) and 89.58, 92.53, 94.21, 95.22, 92.31 % (MnP-I-PVAA) for Sandal-fix Red C_4_BLN, Sandal-fix Turq Blue GWF, Sandal-fix Golden Yellow CRL, Sandal-fix Black CKF, Sandal-fix Foron Blue E_2_BLN, respectively. The pH was in the range of 8.4–9.0 before treatment, reduced after treatment and fall in the range of 6.0–6.9. The dye samples treated by MnP-I-PVAA showed slightly lower pH values versus F-MnP treatments (Fig. 6a).

The COD, BOD and TOC values of textile wastewater considerably higher as compared to synthetic dyes solution and were beyond the permissible tolerance limit set by National Environmental Quality Standards (NEQS). The COD, BOD, TOC were in the range of 1220–2920 mg/L, 650–2015 mg/L and 1050–2450 mg/L, respectively before treatment. However, after treatment the COD, BOD and TOC values significantly reduced (Fig. 6b, c). In case of F-MnP, the reductions in COD, BOD and TOC were in the range of 47–70 %, 41–61 % and 46–65 %, respectively, whereas 52–86 %, 53–76 % and 54–71 % a reductions were observed in MnP-I-PVAA treated samples. The pH of textile wastewater was in the range of 10.0–11.5 before treatment and after treatment reduced considerably and fall in the range of 6.5–7.00. Here, the improvement in water quality assurance parameters was significantly higher, especially in case of PVA–MnP treatment as compared to previous same sort of studies e.g. BOD (330.8 ppm) and COD (370.1 ppm) removal form textile industry effluents using *Irpex lacteus* [36], 70 % COD removal by *P. ostreatus* [37], 75 % COD removal with *P. chrysosporium* and *Coriolus versicolor* [38], 67 % COD removal using *C. versicolo*r [39].

Literature survey revealed that although physicochemical techniques were some time effective, quick and compact, but are not generally employed due to the associated high chemical and operational costs and the biodegradation is good option, especially immobilization to hit the target because in most of the cases, the decolorization and improvement in water quality assurance parameters improvement was found significantly higher in comparison to physicochemical treatments.

### Cytotoxicity reduction

**Fig. 7:**
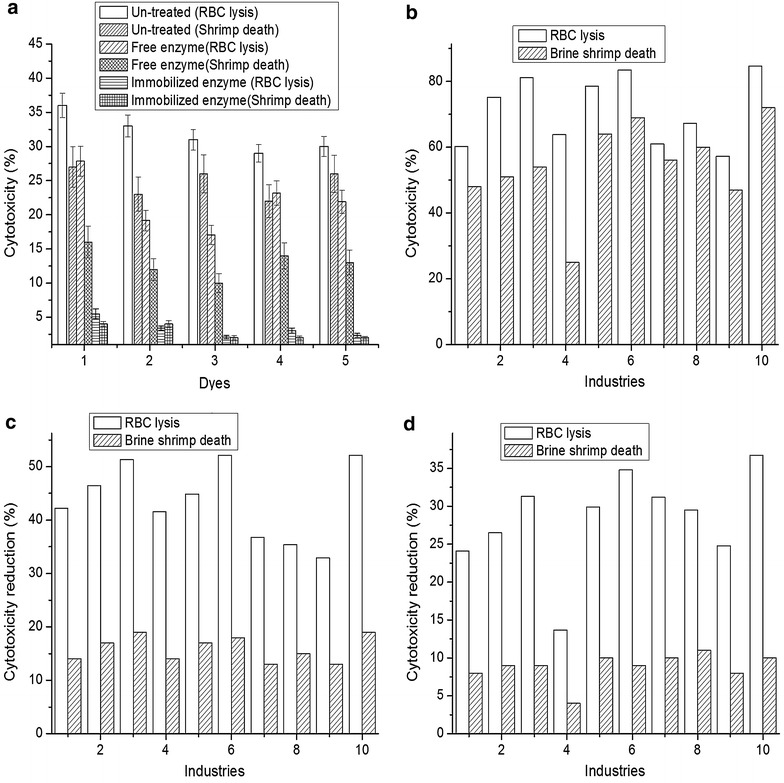
**a** Cytotoxicity (%) of dyes, **b**, **c**, **d** cytotoxicity (%) of textile wastewater (un-treated, F-MnP and MnP-I-PVAA treated)

The cytotoxicity tests (heamolytic and brine shrimp lethality) were performed to check the biological effectiveness of F-MnP and MnP-I-PVA since these tests are used frequently for the toxicity screening of pollutants (air, soil and water) [2]. The order of cytotoxicity of dyes was found in following order before treatment; Sandal-fix Red C_4_BLN (36 %), Sandal-fix Turq Blue GWF (33 %), Sandal-fix Golden Yellow CRL (31 %), Sandal-fix Foron Blue E_2_BLN (30 %) and Sandal-fix Black CKF (29 %) for erythrocyte lysis, whereas it was 27, 23, 26, 26, 22 %, respectively in case of brine shrimp. As shown in Fig. 7a, after treatment the cytotoxicity reduced significantly, both for F-MnP and MnP-I-PVAA. Erythrocytes lysis were 27.85, 19.16 and 17.03 %, 23.17 and 21.89 %, whereas 16, 12, 10, 14 and 13 % were observed in case of brine shrimp for Sandal-fix Red C_4_BLN, Sandal-fix Turq Blue GWF, Sandal-fix Golden Yellow CRL, Sandal-fix Black CKF, Sandal-fix Foron Blue E_2_BLN, respectively for F-MnP treatment. In case of MnP-I-PVAA, the erythrocytes lysis were 5.51, 3.41, 2.07, 3.03 and 2.32 %, whereas 4, 4, 2, 2 and 2 % for brine shrimp of Sandal-fix Red C_4_BLN, Sandal-fix Turq Blue GWF, Sandal-fix Golden Yellow CRL, Sandal-fix Black CKF, Sandal-fix Foron Blue E_2_BLN, respectively. The textile wastewater was also subjected to cytotoxicity testing before and after treatment and results, thus obtained are shown in Fig. 7b–d. Before treatment, the cytotoxicity was considerably higher and found in the range of 22.5–84.6 % (erythrocyte lysis) and 21.0–72 % (brine shrimp lethality) for the wastewater collected from ten textile units. After treatment, cytotoxicity reduced significantly and recorded in the range of 15.2–52.1 % (erythrocyte lysis) and 7.63–19.0 % (brine shrimp lethality) in case of F-MnP, whereas 12.1–36.7 % (erythrocyte lysis) and 3.74–11.0 % (brine shrimp lethality) cytotoxicity range was recorded in case of MnP-I-PVAA treatment. The percentage cytotoxicity reductions of 60.0, 38.2, 36.8, 34.8, 42.8, 37.5, 39.7, 47.3, 42.5 and 38.4 (%) (Erythrocytes lysis) and 86.7, 88.0, 88.9, 93.7, 87.3, 89.2, 83.6, 83.6, 86.0 and 88.2 (%) (Brine shrimp lethality) for textile industries wastewater sample from 1 to 10, respectively were observed for MnP-I-PVAA treatments as compared to un-treated samples. As it was highlighted by Rane et al. [1] that several methods have been used for the decolorization of dyes successfully, however, the toxicity is still a matter of major concern because residues/degradation intermediates and end product might be more toxic than the parent compound. Present study showed that MnP-I-PVAA treatment has the ability to detoxify dyes along with decolorization. Previous few studies also revealed that toxicity may be reduced by biodegradation of treated dyes e.g. 98 % toxicity reduction of industrial effluents was observed, treated by WRF ligninolytic enzymes [40] and similar observations were documented by others [23, 41].

## Conclusions

The MnP produced from *G. lucidum* IBL-05 was immobilized onto PVA beads to explore its efficiency for decolorization and detoxification against set of Sandal reactive dyes (new dyes class) and textile wastewater. The MnP was successfully entrapped into PVAA beads with satisfactory immobilization efficiency and resulting, MnP-I-PVAAB showed exciting outcomes for the decolorization and detoxification of Sandal reactive dyes and textile real wastewater. The efficient dye decolorization and cytotoxicity reduction suggests the feasibility of MnP-I-PVAAB for textile wastewater remediation since bio-remediation is a green technique. Future investigations should be focused on more sensible immobilization techniques along with innovative supports for the improvement of enzyme catalytic properties.
